# Cu-nanoparticles enhance the sustainable growth and yield of drought-subjected wheat through physiological progress

**DOI:** 10.1038/s41598-024-62680-1

**Published:** 2024-06-20

**Authors:** Muhammad Aown Sammar Raza, Jawad Amin, Mohammad Valipour, Rashid Iqbal, Muhammad Usman Aslam, Bilal Zulfiqar, Faqeer Muhammad, Muhammad Arif Ibrahim, Abdullah Ahmed Al-Ghamdi, Mohamed S. Elshikh, Javed Iqbal, Monika Toleikienė, Heba H. Elsalahy

**Affiliations:** 1 Department of Agronomy, Faculty of Agriculture and Environment, The Islamia University of Bahawalpur, Bahawalpur, 63100 Pakistan; 2 Department of Engineering and Engineering Technology, Metropolitan State University of Denver, Denver, CO 80217 USA; 3 Department of Botany and Microbiology, College of Science, King Saud University, P.O. 2455, 11451 Riyadh, Saudi Arabia; 4 Department of Botany, Bacha Khan University, Charsadda, 24420 Khyber Pakhtunkhwa Pakistan; 5 Institute of Agriculture, Lithuanian Research Centre for Agriculture and Forestry, Instituo Al. 1, 58344 Akademija, Kedainiai Lithuania; 6 Leibniz Centre for Agricultural Landscape Research (ZALF), 15374 Müncheberg, Germany

**Keywords:** Wheat, Drought, Cu-nanoparticles, Growth, Wheat production, Biochemistry, Plant sciences

## Abstract

Drought stress (DS) is a significant abiotic stress that limits agricultural productivity worldwide. In semi-arid climates, one potential solution to alleviate the deleterious effects of drought is the use of soil amendments such as nanoparticles. The current research was conducted out to probe the sway of drought at critical growth stages (CGS) of wheat crop (D_0_: Control, D_1_: Drought at tillering stage, and D_2_: Drought at anthesis stage) and the application of Cu-nanoparticles (T_0_: 0 mg L^−1^, T_1_: 300 mg L^−1^, T_2_: 700 mg L^−1^, and T_3_: 950 mg L^−1^) in order to improve drought resilience. Results of the study revealed that DS considerably decreased the wheat growth and yield during CGS. However, Cu-nanoparticles application alleviated the detrimental backlash of DS and led to improvements in various aspects of wheat growth and yield, including plant height, spike length, 1000 grain weight, stomatal conductance, leaf chlorophyll content, water use efficiency, leaf turgor potential, relative water content, and ultimately the grain yield. The use of principal component analysis allowed us to integrate and interpret the diverse findings of our study, elucidating the impact of Cu-nanoparticle treatment on wheat growth and yield under drought. Overall, the study concluded that DS during the anthesis stage had the most significant negative impact on crop yield. However, applying Cu-nanoparticles at the rate of 300 mg L^−1^ proved to be an effective strategy for improving crop productivity by reducing the harmful effects of drought.

## Introduction

World increasing population has a significant impact on agriculture, requiring drought tolerant crops to bridge the gap between food supply and demand. Drought stress (DS) poses a significant threat to global food security, particularly in semi-arid regions^1^. Under this scenario, there is a dire need to develop to such strategies that can increase crop productivity and sustainability having the potential to minimize the ill effects of drought. In recent years, nanotechnology has emerged as a promising field offering novel solutions for sustainable agriculture.

Wheat (*Triticum aestivum* L.) is an important staple food in many regions of the world, including South Asia, America, and Europe. It is called as king of cereals and ranks first among the cereals due to its high nutritional value^2,3^. It has protein, fiber, fat, and carbohydrates content of 9.4–13.9, 1.8–2.3, 1.2–2.5, and 69.1–75.5, g/100, respectively^4,5^. It is a vital source of nutrition for humans and animals, and its production is gradually threatened by drought episodes. Therefore, it is imperative to use modern technologies to enhance wheat yield and drought resilience in order to maintain wheat's status as a staple food^6^.

Drought, the major abiotic stress, not only reduces development and yield, but it also diminishes global crop output^7^. More catastrophic drought periods are projected in the next years, with an increase in average world temperature by 1.5 °C owing to climate variation, which would severely disrupt agricultural production and the farming community^8^. A severe shortage of water can cause damage to plant cell membrane and cell wall architecture, as well as impede cell division and photosynthesis^9^. According to El-Fattah et al.^10^ and Raza et al.^11^, the exposure of crop plants to DS led to a notable decline in photochemical activities and the suppression of enzyme activities involved in the Calvin cycle. Drought and other abiotic stresses can induce considerable changes in seed protein levels and production by disrupting plant development processes^12,13^. DS during critical growth stages (CGS) has been observed to reduce spike length, number of grains per spike (NGPS), and thousand grain weight (TGW) in wheat^14^. However, several studies have published that NPs supplementation under irrigation deficit conditions can have a positive effect on the growth and development of wheat by enhancing nutrient uptake and facilitating metabolic functions^15^.

Nanotechnology involves the study and manipulation of atomic or molecular aggregates with sizes ranging from 1 to 100 nm as describe by Rasheed et al.^16^. Nanoparticles (NPs) distinct physiochemical characteristics, such as their smaller size and large surface area, have led to their extensive application in various disciplines within the field of biosciences^15^. Notably, NPs made of aluminum (Al), cesium oxide (Ce_2_O_3_), copper (Cu), gold (Au), magnetized iron (Fe), silver (Ag), silica (Si), titanium dioxide (TiO_2_), zinc (Zn), and zinc oxide (ZnO) have proven useful in agriculture^16,17^. NPs offer potential benefits in different aspects of agriculture, such as enhancing crop production, protecting plants, improving crop quality, and optimizing fertilizer and irrigation practices^16^. The utilization of NPs in the field of crop sciences has been progressively growing; leading to the identification of several beneficial impacts on crop plants^18^. In particular, NPs treatment has been found to increase spike length (SL), NGPS, and TGW^19^. These positive effects are attributed to the creation of growth-regulating substances by NPs, such as indole-3-acetic acid and cytokinins, which enhance plant growth hormones, source-sink relationship, and ultimately lead to better grain weight^20,21^. Venkatachalam et al.^22^ found that zinc oxide NPs application resulted in favorable growth and reduced fertilizer applications by half in cotton. Positive effects of different NPs application on the germination, growth, physiological activities, water and fertilizers use efficiency, root growth, branching, biomass, and photosynthetic pigments have also been reported^23,24^. Do Espirito Santo Pereira et al.^25^ found that seed priming with NPs improved nutrient use efficiency, enhanced photosynthetic activity, and improved grain quality in wheat. Nanotechnology has the potential to significantly impact various scientific and biological fields. In particular, it is anticipated that nanotechnology will play a vital role in revitalizing agriculture and emerge as a strong economic force in the coming years^18^. Convincingly, a nanotech-based agricultural reorientation may enhance quality food production while conserving resources and the environment. NPs may revolutionize crop science research and transform agriculture into an industry by exploring their comprehensive application profile.

Copper (Cu) is an important micronutrient required in very minute quantities by plants, and is identified in improving chlorophyll formation, plant growth, development, and yield^26^. It is involved in various enzyme systems regulating many biochemical reactions in plants essential for photosynthesis, respiration and metabolism of proteins and carbohydrates^27,28^. Copper nanoparticles (Cu-NPs) are distinguished for their significant physiological advancement in regulating stomatal activity and reducing water loss under DS^23,29^. Cu-NPs application has been reported to enhance seed yield and quality, as well as drought resilience in soybean^30^. Furthermore, Van Nguyen et al.^23^ reported an increase in the chlorophyll content, enzyme activity, total seed number, and grain yield of maize under drought conditions by the application of Cu-NPs. However, limited research has been published to explore the potential of Cu-NPs in boosting wheat yield under drought conditions. To explore the myriad advantageous characteristics of Cu-NPs in enhancing the drought resilience and production potential of wheat, this study was designed with the objective to assess the effect of Cu-NPs on the growth, yield and physiological attributes of drought-subjected wheat.

## Materials and methods

### Experimental location and crop husbandry

**Figure 1:**
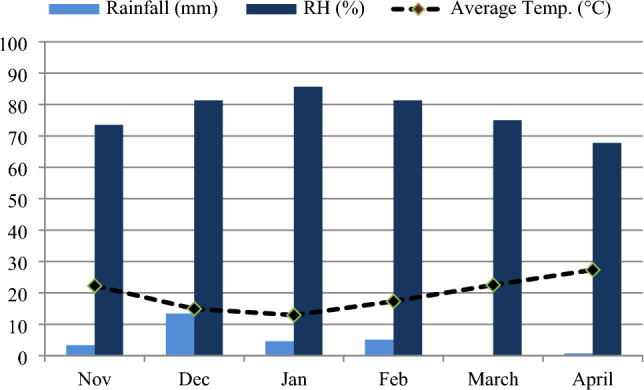
Data on relative humidity (RH), average rainfall and average temperature throughout the wheat growing season.

The experimental trial was carried out at the research area of Agronomy department, The Islamia University of Bahawalpur, Pakistan situated at longitude: 71°40′59.99″E and latitude: 29°23′60.00″N. The experimental design consisted of a randomized complete block design (RCBD) with four replications in a factorial layout. Four treatments of Cu-NPs were administered at every studied growth stage as follows: T_0_ = control, T_1_ = Cu-NPs @ 300 mg L^−1^, T_2_ = Cu-NPs @ 700 mg L^−1^, T_3_ = Cu-NPs @ 950 mg L^−1^. The tillering (TS) and anthesis (AS) stages were subjected to DS, with full irrigation serving as the control. The acquisition of the seeds of Galaxy 2013 (wheat variety) was made from the Regional Agricultural Research Institute, Bahawalpur, ensuring originality and authenticity. After sterilizing the seeds with 70% ethanol for one minute and 3% sodium hypochlorite (NaClO) for thirty minutes, they were rinsed five times with distilled water. On November 12th, 2019, seeds were sown in plastic pots measuring 26 × 29 cm, filled with 16 kg soil that had been supplemented with NPs. The experimental soil's physiochemical analysis is presented in Table 1, while Fig. 1 displays the data concerning relative humidity, average rainfall, and temperature of the wheat growing season. A transparent plastic covering was positioned above the wire-house for shielding the crop from precipitation whenever necessary. All the pots received equal watering till complete emergence.

**Table 1:** Experimental soil's physicochemical properties.

Properties	Soil profile
Available potassium (ppm)	112
Available phosphorus (ppm)	6.75
Ammoniac N (mg g^−1^)	1.58
Electric conductivity (dS m^−1^)	2.55
Organic matter (%)	0.92
pH	7.23
Clay (%)	11
Silt (%)	33.5
Sand (%)	61
Texture class	Sandy loam soil

### Drought imposition and characterization of Cu-NPs

Drought stress was imposed at tillering (TS) and anthesis (AS) stages, whereas, normal supply of water was used as control treatment. All pots received the same amount of water at 85% of soil water holding capacity (WHC), also referred as control treatment, until the commencement of DS. After that, DS was enacted at TS and AS by maintaining the soil WHC at 30%.

**Figure 2:**
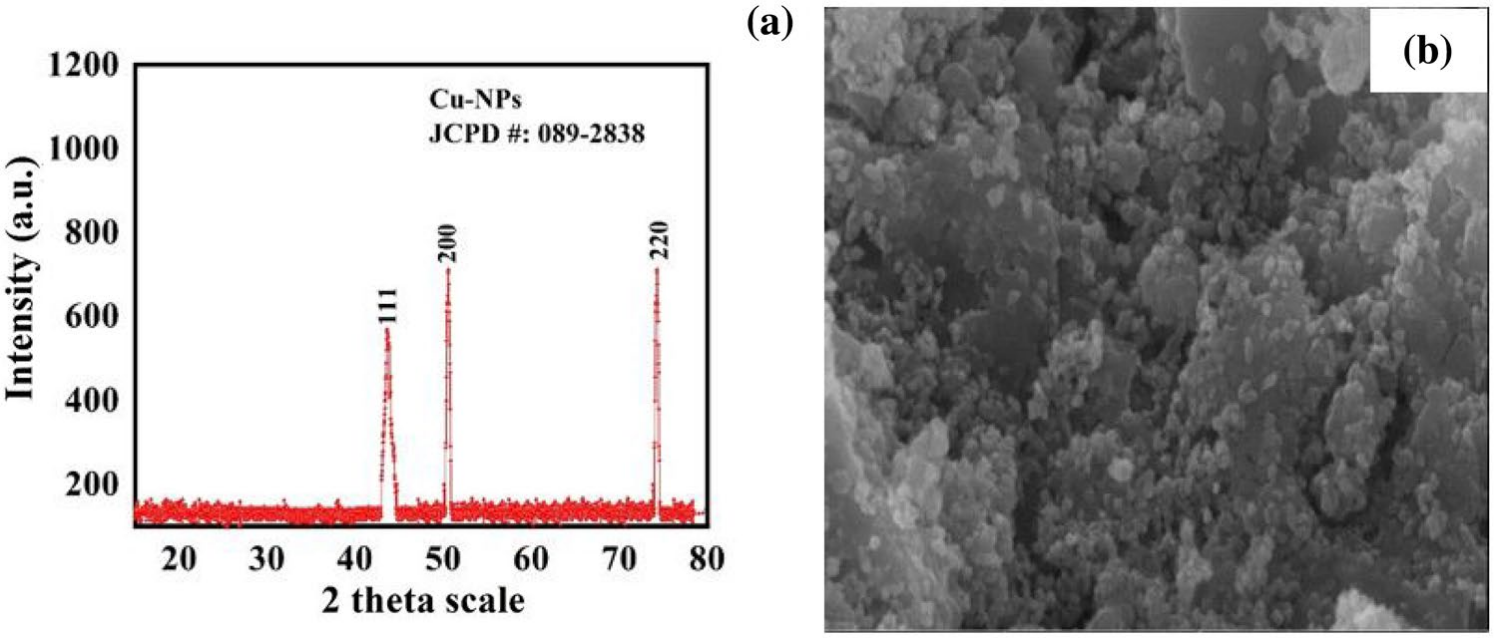
The XRD analysis (**a**) to determine the crystalline nature and scanning electron microscope analysis (**b**) to determine the shape of Cu-NPs.

Cu-NPs were obtained from Sigma Aldrich Company, USA. The X-ray diffraction spectroscopy (XRD) test was performed to analyse the crystalline nature and purity of Cu-NPs. The typical size of the Cu-NPs was observed to be 21.86 nm using Debye Scherrer's equation. The findings of the XRD study of Cu-NPs are shown in Fig. 2a which indicates that the Cu-NPs are completely pure and crystalline with a uniform structure as described by Betancourt-Galindo et al.^31^ and Phul et al.^32^. Furthermore, scanning electron microscope (SEM) analysis was performed to determine the shape and topology of the Cu-NPs. Figure 2b determined the SEM image of Cu-NPs and demonstrates that the Cu-NPs have a round spherical shape as reported earlier by Mali et al.^33^.

### Plant height and yield-related parameters

The plant height (cm) and yield related parameters i.e., SL (cm), NGPS, TGW (g), biological (BY) and grain yield (GY) plant^−1^ (g), and harvest index (HI) were measured as per established protocols/procedures.

### Water use efficiency and leaf chlorophyll contents

Hussain and Al. Jaloud^34^ proposed a formula to calculate the water use efficiency (WUE, g pot^−1^ mm^−1^) given as under:

$${\text{WUE}} = {\text{Grain }}\;{\text{Yield}}/{\text{Total }}\;{\text{Water }}\;{\text{Applied}}$$

A chlorophyll meter (CL-01, USA) was used for the measurement of leaf chlorophyll contents.

### Water relations

The water potential of leaf (-MPa) was measured by employing a portable water potential tool (Chas W. Cook Div., made up of England) on the completely extended freshest leaf, specifically the fourth leaf from the top.

The calculation of excised leaf water loss (ELWL) and relative water content (RWC) was performed according to the formulae described by Haider et al.^14^.

$${\text{ELWL }}\left( {\text{\% }} \right) = \frac{{{\text{FW}} - {\text{WW}}}}{{{\text{DW}}}}\quad {\text{RWC }}\left( {\text{\% }} \right) = \frac{{{\text{FW}} - {\text{DW}}}}{{{\text{TW}} - {\text{DW}}}} \times 100$$

Here FW represents fresh weight, WW denotes wilted weight, DW represents dry weight, and TW stands for turgid weight.

To determine leaf turgor potential (TP, MPa) the following equation was used;

$$\text{TP}=\text{WP}-\text{OP}$$

where WP = water potential (ψ_w_) and OP = osmotic potential (ψ_s_).

### Stomatal conductance

Measurements of stomatal conductance (mmol of H_2_O m^−2^ s^−1^) were recorded using an automated equipment MK-3, Hertford, Herts (Delta-T Devices, Burwell Cambridge, made up of England).

### Statistical analysis

STATISTIX (version 8.1) software was employed to do the analysis of variance and compare the mean values at the 5% probability level using the least significant difference (LSD) technique, as stated by Sharma^35^. A PCA (principal component analysis) was also done on the data, and the findings were visualized using a biplot graph based on the PCA main components (PC 1 and PC 2).

### Ethics approval and consent to participate

This study does not include human or animal subjects.

### Statement on guidelines

All experimental studies and experimental materials involved in this research are in full compliance with relevant institutional, national and international guidelines and legislation.

## Results

### Growth and yield parameters

Drought stress (DS) significantly affected all the growth, yield and related parameters (SL, NGPS, TGW, HI) of wheat (Table 2). All the aforementioned parameters were considerably decreased when DS was enacted at TS and AS, while higher values of PH, SL, NGPS, TGW, BY, GY and HI were noted in control treatment (D_0_). However, the application of Cu-NPs significantly mitigated the adverse effects of drought and improved the studied parameters of wheat both under control and DS conditions. An increase in PH, SL, NGPS, TGW, BY, GY and HI was observed when Cu-NPs were applied @ 300 mg L^−1^ at TS and AS, in comparison to control treatment. However, higher doses of Cu-NPs (700 and 950 mg L^−1^) reduced all these parameters when compared with control treatment (T_0_) as presented in Table 2.

**Table 2:** Plant height and yield related traits of wheat as affected by Cu-NPs under drought.

Treatments		Parameters						
Drought	Cu-NPs	Plant height	Spike length	Number of Grains/Spike	1000-grain weight	Biological yield	Grain yield	Harvest index
D_0_ = Control	T_0_	66.12b	13.4ab	36.66a	29.41b	16.77b	10.56b	52.51b
	T_1_	68.13a	13.56a	35.33b	30.26a	17.2a	11.71a	55.46a
	T_2_	65.43bc	13.16b	34.66c	28.81c	15.52bc	10.41c	50.24c
	T_3_	63.76d	13.06c	34 0.56c	28.41c	15.2c	10.26c	49.54d
D_1_ = Drought at tillering	T_0_	65.16b	12.96b	35.66a	28.22b	15.88b	9.17b	53.45a
	T_1_	65.5a	13.53a	34.33b	29.07a	16.3a	10.32a	50.34b
	T_2_	64.03c	12.83b	33.66c	27.62c	14.6bc	9.02b	48.12c
	T_3_	63.43d	12.66c	33.0 cd	27.22d	14.38c	8.87c	47.42d
D_2_ = Drought at anthesis	T_0_	61.76b	12.8b	35.06a	27.01b	14.78b	7.96b	50.5 7a
	T_1_	62.43a	13.36a	33.66b	27.86a	15.26a	9.11a	47.45b
	T_2_	59.73c	12.53c	33.21bc	26.41c	13.53c	7.81c	45.23c
	T_3_	59.26c	12.26d	32.33c	26.0 cd	13.28c	7.66d	44.53d

T_0_, T_1_, and T_2_, T_3_ denotes control, 300 mg L^−1^, 700 mg L^−1^, and 950 mg L^−1^ Cu-NPs, respectively. The means that are sharing similar letter case does not exhibit significant differences at 5% level of probability.

### Water use efficiency (g pot^−1^ mm^−1^) and leaf chlorophyll contents (%)

**Figure 3:**
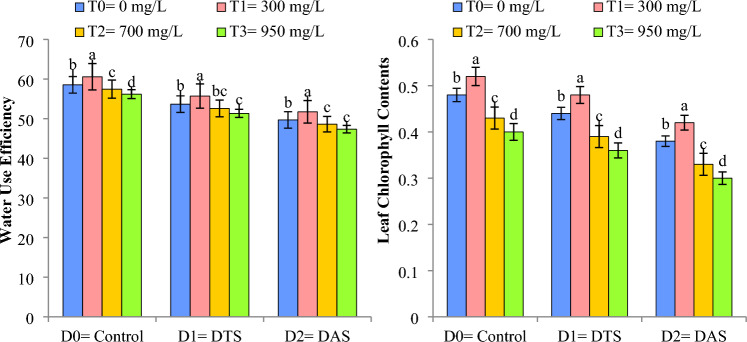
Impact of Cu-NPs application on the WUE and leaf chlorophyll contents of wheat under drought. D_0_: control, D_1_: drought at tillering stage (DTS), D_2_: drought at anthesis stage (DAS). The error bars denote the standard error (n = 4).

Cu-NPs application at CGS (TS and AS) demonstrated enhanced WUE and leaf chlorophyl contents (LCC) of wheat during water deficit conditions. Under DS conditions, the WUE and leaf chlorophyl contents shows a decline during the TS (D_1_) and AS (D_2_), respectively, when compared with control treatment (D_0_). Cu-NPs (300 mgL^−1^) significantly reduced the drought impact and increased the WUE and LCC by 3.78% and 10%, respectively, in comparison to control treatment (T_0_), as depicted in Fig. 3.

### Stomatal conductance (mmol m^−2^ s^−1^)

**Figure 4:**
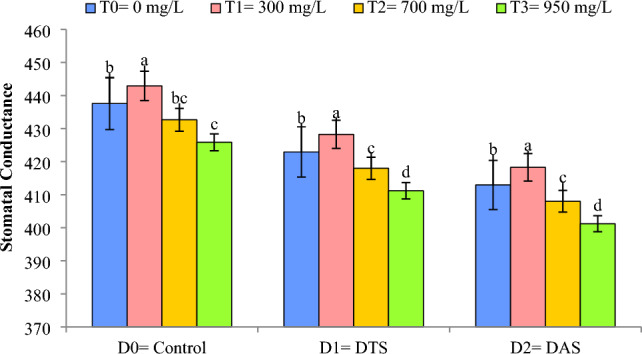
Impact of Cu-NPs application on the SC of wheat under drought. D_0_: control, D_1_: drought at tillering stage (DTS), D_2_: drought at anthesis stage (DAS). The error bars denote the standard error (n = 4).

Figure 4 illustrated that DS negatively affected the stomatal conductance (SC) of wheat. During DS the SC shows a decline during the TS and AS, in comparison to the control treatment. Cu-NPs application at CGS demonstrated enhanced wheat WUE during water scarce (D_1_ and D_2_) and control (D_0_) conditions. Maximum values of SC (442.93 mmol m^−2^ s^−1^) were noted by the application of Cu-NPs (300 mg L^−1^) under control conditions (D_0_) and minimum values for SC (401.21 mmol m^−2^ s^−1^) were observed when Cu-NPs were applied @ 950 mg L^−1^ (T_3_) at AS (D_2_).

### Leaf relative water contents (%) and excised leaf water loss (%)

**Figure 5:**
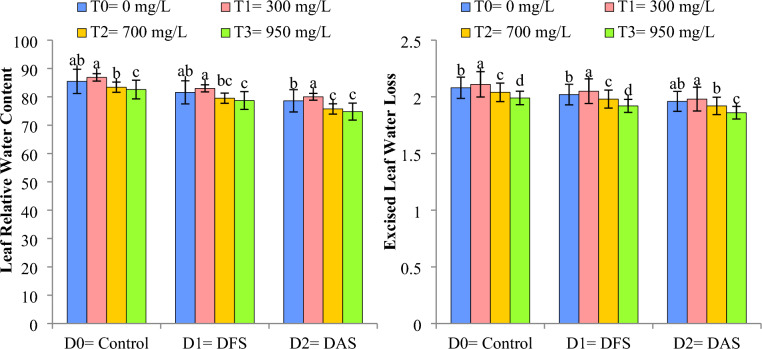
Impact of Cu-NPs application on the LRWC and ELWL of wheat under drought. D_0_: control, D_1_: drought at tillering stage (DTS), D_2_: drought at anthesis stage (DAS). The error bars denote the standard error (n = 4).

The application of Cu-NPs significantly improved the leaf relative water contents (LRWC) and excised leaf water loss (ELWL) in wheat both under control and DS situations as depicted in Fig. 5. Reduction in LRWC and ELWL was recorded when DS was imposed at AS while maximum values for both parameters was noticed in under normal irrigation (D_0_). However, Cu-NPs when applied @ 300 mg L^−1^ (T_1_) resulted in an increase in LRWC and ELWL followed by T_0_ and reduced values for LRWC and ELWL were recorded when Cu-NPs were applied @ 950 mg L^−1^ (T_3_) as compared to control treatment (T_0_).

### Leaf water potential (-MPa) and Leaf turgor potential (MPa)

**Figure 6:**
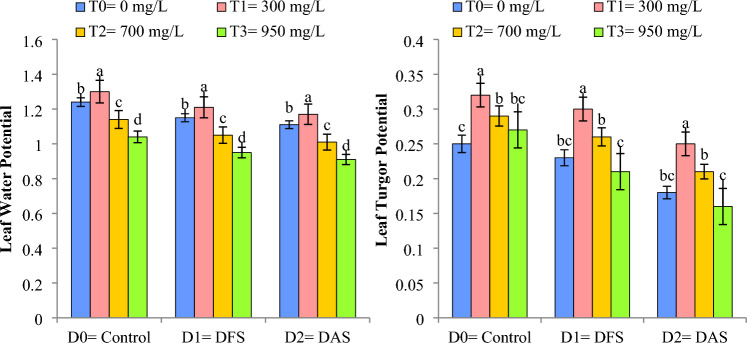
Impact of Cu-NPs application on the LWP and LTP of wheat under drought. D_0_: control, D_1_: drought at tillering stage (DTS), D_2_: drought at anthesis stage (DAS). The error bars denote the standard error (n = 4).

Significant reduction in leaf water potential (LWP) and leaf turgor potential (LTP) of wheat were observed under DS circumstances. DS reduced the LWP and LTP when imposed at TS (D_1_) and AS (D_2_) in comparison to control (D_0_). The application of Cu-NPs @ 300 mg L^−1^ significantly reduced the drought impact on LTP and LWP by 18% and 5.6%, respectively, when compared to control conditions. Higher values of LWP and LTP were recorded in T_1_ followed by T_0_ under control conditions (D_0_), whereas, reduction in both parameters was noticed in T_3_ when drought was imposed at AS (D_2_) as depicted in Fig. 6.

### Principal component analysis

**Figure 7:**
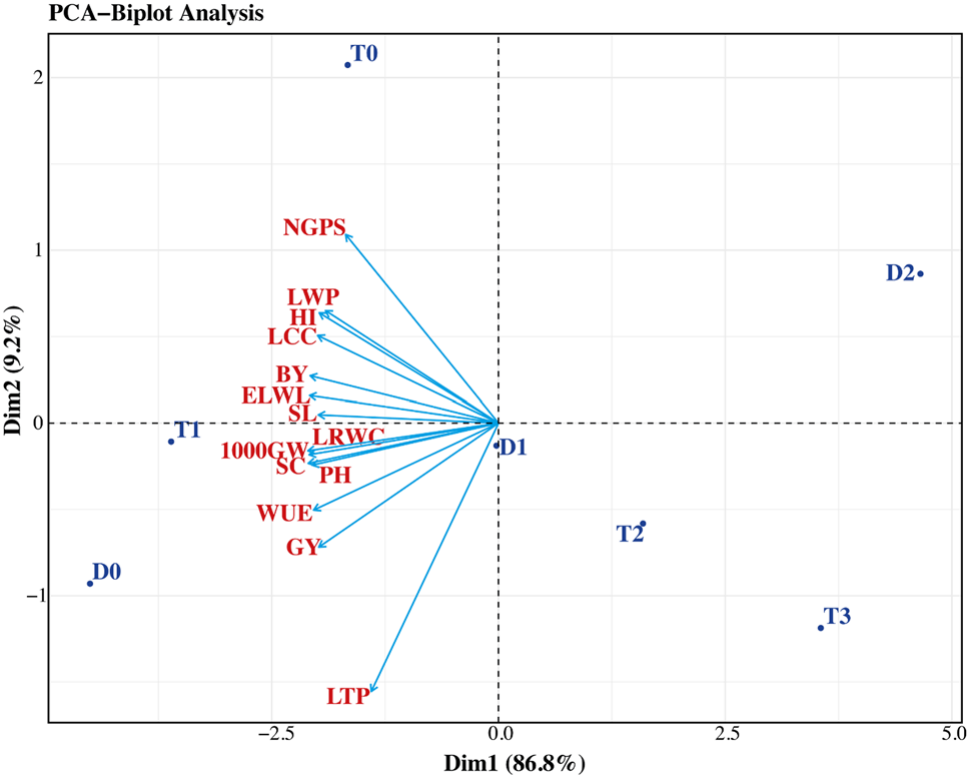
PCA of morpho-physiological attributes of wheat crop under Cu-NPs and drought. D_0_: control, D_1_: drought at TS, D_2_: drought at AS and T_0_, T_1_, T_2_, and T_3_ signposts control, 300 mg L^−1^, 700 mg L^−1^, and 950 mg L^−1^ Cu-NPs, respectively.

The variability and relationships between the morpho-physiological characteristics of wheat under various drought and Cu-NPs treatments were investigated using principal component analysis (PCA) as depicted in Fig. 7. Results revealed that the first two PCs captured 96% of the total variations. Out of these PCs the PC1 and PC2 captured 86.8% and 9.2% variations. Variation in PC1 is mainly due to PH, SL, NGPS, TGW, GY, BY, HI, LWP, LRW, LCC, WUE, SC, and ELWL. While in PC2 main variation was due to LTP. Our results demonstrated that T_1_ (300 mg L^−1^) is better because it is clustered near the origin.

## Discussion

Drought stands out as a primary obstacle to achieving sustainable crop production^11,36^. Significant decrease in the growth and yield of wheat during drought underscore the need to develop and identify strategies to alleviate its impact. The current study demonstrate that the application of Cu-NPs significantly influences the growth, yield, and related factors under both normal and drought conditions. These results align with previous findings suggesting that application of Cu-NPs can effectively mitigate drought stress, leading to improved wheat growth and yield, especially under controlled conditions^37^.

Drought stress significantly reduced the chlorophyll contents in wheat due to the over production of reactive oxygen species (ROS) that leads to lipid peroxidation and consequently chlorophyll destruction^11,38,39^. Similarly, Gill and Tuteja^40^ and Aslam et al.^7^ described that DS decreased the chlorophyll contents in wheat and quinoa, respectively, which are in line with our findings. However, Cu-NPs improved the leaf chlorophyll contents when applied in lower concentration (300 mg L^−1^) as it increases cytokinin’s production that increases the metabolic activity and cell growth and reduces the ROS production and so helps to enhance chlorophyll contents^41,42^ whereas, higher concentrations (700 mg L^−1^, 950 mg L^−1^) reduced the chlorophyll contents by decreasing photosynthetic performance and antioxidant activities^16,43^.

WUE decreased under DS when imposed at CGS, i.e., tillering (TS) and anthesis (AS) stages. Likewise, Muhammad et al.^42^ and Raza et al.^44^ confirmed that DS decrease the WUE in wheat. Cu-NPs application helps to improve plant functions such as water availability and glycolysis metabolism and hence the WUE in wheat crop as also reported by Elshayb et al.^41^ and Raza et al.^44^. Figure 7 indicates that WUE is a significant determinant for wheat yield vacillations at critical growth stages.

Stomatal conductance (SC) is an important parameter to explore the drought resilience in a crop. DS abridged the water uptake from the soil which ultimately leads to stomatal closure and hence reduced the SC as perceived in current study. Similar outcomes have also been reported in wheat by Haider et al.^14^ and Raza et al.^45^. Lower concentration of Cu-NPs improved the SC both under control and DS conditions. Likewise, Raza et al.^44^ reported an increase in SC by the application of NPs in wheat under DS conditions because NPs increase the production of phytohormones, accumulation of osmolytes, improve water uptake and photosynthetic efficiency thus offering better resistance to wheat crop against water deficit conditions^16,46^.

Limited water availability significantly decreased the LRWC, ELWL, LWP and LTP at TS and AS in comparison to control treatment. Similar findings have been reported by others under limited water supply probably due to reduced soil moisture, cell division SC, and disability of plants to nutrient absorption^11,41,42^. Cu-NPs application improved the aforementioned attributes both under control and irrigation deficit situations. The findings of our study are in-line with others who reported that NPs improved the water relations (LRWC, ELWL, LWP and LTP) as the application of NPs increases the root biomass, lateral roots formation and hormonal signaling, thereby improving water uptake and maintaining better water relations under DS conditions^47,48^.

Significant reduction in plant height was noticed under water deficit conditions when compared with control treatment. Similarly, Raza et al.^2^ and Raza et al.^11^ reported a decrease in plant height of wheat and quinoa, respectively, under DS due to the dehydration of plant cell, low turgidity and reduced cell division. However, application of Cu-NPs in low concentration (300 mg L^−1^) improved the plant height of wheat crop by improving the source sink relationship, water and nutrient uptake and photosynthetic activity^44^. Similarly, Ahmed et al.^38^ and Raza et al.^11^ reported a positive effect of NPs application on plant height of rice and wheat under both control and DS conditions, respectively.

The yield contributing attributes (SL, NGPS, TGW), biological yield, grain yield and harvest index were significantly affected under water deficit conditions. The decrease in SL, NGPS, TGW and grain yield was found to be more pronounced as DS was imposed at anthesis stage. Raza et al.^11^ and Muhammad et al.^42^ observed similar outcomes that DS reduce the yield contributing attributes in wheat and quinoa, respectively, due to decrease in plant metabolic processes resulting from insufficient water availability, inadequate absorption and distribution of photosynthates^16^. Likewise, Aslam et al.^7^ and Raza et al.^45^ also reported a reduction in grain yield and associated parameters in quinoa and wheat, respectively, under short supply of water. The application Cu-NPs significantly improved the yield components, biological and grain yield both under control and drought conditions. The lower concentration of Cu-NPs (300 mg L^−1^) significantly enhanced the aforementioned yield components and hence the biological and grain yield as NPs improves water and nutrient uptake, photosynthetic efficiency, osmolytes accumulation, antioxidant activities, and gene expression and attenuated the adverse effects of drought stress^49,50^. Similarly, Yasmeen et al.^19^ reported an increase in SL, NGPS and grain yield of wheat by the application of Cu-NPs under DS conditions. However, the higher concentration of Cu-NPs (700 mg L^−1^, 950 mg L^−1^) negatively affected the growth, yield and yield components as the excessive use of NPs cause oxidative stress and physiological abnormalities in plants, resulting in decreased antioxidant activities and gas exchange characteristics^16,50^. Similar findings about the toxicity of NPs in plants have been reported earlier by Wang et al.^43^ and Hou et al.^51^. The biplot graph of PCA revealed several significant factors, including SC, leaf chlorophyll contents and WUE, as key explanatory variables contributing to resilience of wheat to drought, consistent with findings of Aslam et al.^7^ (Fig. 7). Concludingly, the anthesis stage of wheat was found most sensitive to DS due to limited water availability and compromised nutrient transport to developing seeds, resulting in reduced seed size, and ultimately lowering overall wheat yield. It's worth noting that only the appropriate amount of Cu-NPs shows a positive response, while excessive levels of Cu-NPs can induce toxicity by disrupting plant metabolic processes, triggering oxidative stress and cellular damage, inhibiting photosynthesis, impairing nutrient uptake and transport, ultimately inhibiting growth and reducing the yield of wheat^16,23,50^.

## Conclusion

DS severely abridged the wheat growth, water relations, physiological attributes, and yield when administered at CGS. The application of NPs reduced the negative consequences of DS by enhancing water and nutrient retention capacity and fertility of soil, and the source to sink relationship. Under both control and drought conditions, the application of Cu-NPs enhanced wheat growth, WUE, physiological parameters, water-related characteristics, and yield. Furthermore, the AS seems to be more vulnerable stage to drought, and Cu-NPs (300 mg L^−1^) soil treatment is the most effective strategy for mitigating the deleterious sways of drought and to upsurge the productivity of wheat under controlled pot conditions. Therefore, further field trials are necessary to validate the applicability and effectiveness of Cu-NPs in improving drought resilience in wheat under real-world agricultural settings.

## Data Availability

The datasets analysed during this study are included in this manuscript.
